# Low ankle-brachial index and cognitive function after stroke—the PROSpective with Incident Stroke Berlin (PROSCIS-B)

**DOI:** 10.3389/fneur.2022.963262

**Published:** 2022-09-28

**Authors:** Maria R. V. Stillfried, Pia S. Sperber, Leonie H. A. Broersen, Shufan Huo, Sophie K. Piper, Peter U. Heuschmann, Matthias Endres, Bob Siegerink, Thomas G. Liman

**Affiliations:** ^1^Center for Stroke Research Berlin, Charité – Universitätsmedizin Berlin, Berlin, Germany; ^2^German Centre for Cardiovascular Research DZHK, Charité –Universitätsmedizin Berlin, Berlin, Germany; ^3^Klinik und Hochschulambulanz für Neurologie, Charité- Universitätsmedizin Berlin, Berlin, Germany; ^4^German Center for Neurodegenerative Disease DZNE, Berlin Charité-Universitätsmedizin Berlin, Berlin, Germany; ^5^Institute of Biometry and Clinical Epidemiology, University of Würzburg, Würzburg, Germany; ^6^German Center for Neurodegenerative Disease DZNE, partner site Berlin Charité-Universitätsmedizin Berlin, Berlin, Germany; ^7^NeuroCure Clinical Research Center, Charité – Universitätsmedizin Berlin, Berlin, Germany; ^8^Department of Clinical Epidemiology, Leiden University Medical Center, Leiden, Netherlands; ^9^Department of Neurology, Carl von Ossietzky University of Oldenburg, Oldenburg, Germany

**Keywords:** ankle-brachial index, ischemic stroke, outcome, post stroke cognitive function, atherosclerosis

## Abstract

**Introduction:**

Low ankle-brachial index (ABI) ≤0. 9 is a marker for generalized atherosclerosis and a risk factor for cognitive decline in the general population.

**Objective:**

To evaluate the impact of ABI ≤0.9 on cognitive function up to 3 years after first-ever ischemic stroke.

**Methods:**

Data was used from the “PROspective Cohort with Incident Stroke-Berlin” (PROSCIS-B; NCT01363856). ABI was measured at baseline and categorized into normal (1.4–0.9) vs. low (≤0.9). Cognitive function was assessed with the Montreal Cognitive Assessment (MoCA) and the Mini-Mental-State-Examination (MMSE) at baseline and with the Telephone Interview for Cognitive Status-modified (TICS-m) at 1–3 years of follow-up. We performed confounder adjusted generalized linear models (GLM) to calculate relative risks (RR) for cognitive impairment at baseline (MMSE≤26; MoCA≤25) and linear mixed models (LMM) to estimate the impact of low ABI on TICS-m over time.

**Results:**

We included 325 patients [mean age: 66 (SD = 13); 38% female, median NIHSS = 2 (IQR = 1–4), ABI≤0.9: 59 (18%)]. Patients with low ABI were at increased risk of cognitive impairment at baseline (adjusted RR for MoCA≤25 = 1.98; 95%-CI:1.24 to 3.16). TICS-m scores were consistently lower over time in patients with low ABI (adjusted ß = −1.96; 95%-CI:−3.55 to −0.37). Independent of ABI, cognitive function did not decline over time (adjusted ß:0.29; 95%-CI:−0.06 to 0.64).

**Conclusion:**

In patients with mild to moderate first-ever ischemic stroke, low ABI is associated with reduced cognitive function over a 3-year follow-up.

**Study Registration:**

https://clinicaltrials.gov; Unique identifier: NCT01363856.

## Introduction

The ankle-brachial index (ABI) is a reliable, non-invasive, and inexpensive screening tool for peripheral artery disease (PAD) (1, 2). Low ABI ≤ 0.9 is a sensitive marker for generalized atherosclerosis and independently associated with cognitive impairment and decline in the general population (3, 4).

However, observational studies investigating the impact of low ABI on cognition after stroke are limited, particularly regarding long-term cognitive outcome (5). Stroke occurrence in itself is associated with ensuing cognitive impairment and decline (6). Between 20 and 80% of stroke survivors suffer from cognitive deficits, whereby the severity and recurrence of stroke are related to subsequent cognitive decline (6, 7). Low ABI could be a useful marker to detect cognitive impairment as a stroke-related disability and monitor secondary prevention therapies. A cross-sectional study of 103 hospitalized patients with acute lacunar stroke found that low ABI is associated with cognitive impairment in the acute stroke phase using the Montreal Cognitive Assessment (MoCA; cutoff score at ≤23) (5). Nonetheless, data on low ABI and cognitive function over time after ischemic stroke are lacking.

## Aim

We aimed to investigate whether low ABI ≤ 0.9 is associated with reduced cognitive function at baseline and subsequent decline over a three-year follow-up after first-ever ischemic stroke.

## Methods

### Study design and population

This study is part of the Prospective Cohort with Incident Stroke Berlin (PROSICS-B), an observational, hospital-based prospective cohort study, described in detail elsewhere (8). Between January 2010 and May 2013 stroke patients were recruited at three stroke units of the Charité – Universitätsmedizin Berlin within 7 days after stroke onset (see flow chart in Supplementary Figure 1). Inclusion criteria were patients with (1) first-ever ischemic stroke, primary intracranial hemorrhage, or cerebral venous sinus thrombosis, (2) aged 18 and older, (3) written informed consent by patient or legal guardian prior to study participation. Exclusion criteria were (1) prior stroke (definition according to WHO criteria), (2) patients with brain tumor or brain metastasis, (3) participation in an interventional trial.

Patients were followed up annually with telephone-based interviews for up to 3 years. Patients who had suffered a prior stroke, presented brain tumors, brain metastases or participated in an intervention study were excluded. Only patients with mild to moderate ischemic stroke events, defined by a score of < 16 in the National Institutes of Health Stroke Scale (NIHSS), were included in the analysis. The assessment of ABI and MoCA were introduced to the study as an amendment in January 2011, ~1 year after patient enrollment had started according to the requirements of the ethical committee.

### Patient characteristics

Baseline characteristics were assessed upon admission, including sociodemographic characteristics (e.g., age, sex and education, living situation and lifestyle habits), laboratory blood measures, stroke severity, and cardiovascular risk factors (BMI, current smoking and alcohol consumption, history of hypertension, diabetes mellitus and coronary heart disease).

### ABI measurement

ABI was measured using a manual cuff and a portable Doppler ultrasound device. Participants were assessed in supine position following a 5-min resting period. The systolic blood pressure was determined for the brachial arteries, posterior tibial and dorsal pedis arteries bilaterally. Measurements were assessed at all three stroke centers following a pre-established standard operating procedure.

In accordance with the guidelines issued by the American Heart Association, ABI was calculated on each side by dividing the higher value of the posterior tibial or dorsal pedis artery systolic blood pressure by the higher systolic blood pressure of both brachial arteries (9). We used the lower ABI value. Low ABI was defined as ≤ 0.9. Participants with abnormally high ABI ≥ 1.4, indicative of non-compressible blood vessel calcification, were excluded (9).

### Outcome definitions

Our outcome of interest was cognitive function after stroke at baseline and over a three-year follow-up period. Cognitive function at baseline was assessed using the Montreal Cognitive Assessment (MoCA) and the Mini Mental State Examination (MMSE). Cognitive impairment was defined as a MoCA score ≤ 25 or a MMSE score ≤ 26 (10–12). Cognitive function over time was measured with the Modified Telephone Interview for Cognitive Status (TICS-m), a screening instrument for cognitive dysfunction with 20 items and a maximum score of 50, which was administered annually *via* telephone interview over a three-year follow-up (13).

### Statistical methods

Generalized linear models (GLM) were used to calculate crude and confounder adjusted relative risks (RR) for cognitive impairment at baseline (MoCA ≤ 25; MMSE ≤ 26) in patients with low versus normal ABI and corresponding 95% confidence intervals (CI). We performed linear mixed models (LMM) to calculate effect sizes (ß) and corresponding 95% CI for the crude and confounder adjusted association between low vs. normal ABI and cognitive function over a three-year follow-up, using the annual TICS-m score.

To explore whether the severity of low ABI had an effect on cognitive function in the sense of a dose-response relationship, we further categorized low ABI into mildly low (0.75–0.9) and moderately to severely low ABI (< 0.75) and recalculated our main analyses with these subgroups compared to normal ABI.

Multiple models were used to control for possible sources of confounding. Potential confounding factors were selected a priori according to their presumable impact on both the exposure (ABI) and the outcome (cognitive function). Model 1 adjusted for the sociodemographic variables age (continuous), sex and years of education received (≤ 10 years of schooling, > 10 years of schooling). Model 2 additionally adjusted for the cardiovascular risk factors BMI (kg/m^2^), current smoking (yes/no), history of diabetes mellitus (yes/no) and hypercholesterolemia (yes/no), and history of coronary heart disease (yes/no) and atrial fibrillation (yes/no). Model 3 moreover adjusted for stroke severity as defined by the NIHSS (in 2 categories: 0–4 and 5–15).

We conducted different sensitivity analyses, described in Methods I of the Supplementary material.

Data were prepared using IBM Statistics for Windows, version 25 (IBM Corp, Armonk, NY). All descriptive and statistical analyses were calculated using Stata version 14.1 (Stata Corp, College Station, TX). Data visualizations were performed in the R project (R 4.0.0) using the ggplot2 package.

### Ethics approval

Patients or their legal guardians gave written informed consent prior to study participation. The study was approved by the Charité – Universitätsmedizin Berlin ethics committee (EA1/218/09) and was conducted according to the declaration of Helsinki.

## Results

### Study population

This analysis included 325 patients with mild to moderate ischemic stroke (NIHSS score < 16) and data on ABI (for detailed information on patient inclusion and exclusion see the flow chart in Supplemental Figure 1).

Mean age at study enrollment was 66 years (SD, 13), 38% (*n* = 123) were female. Median NIHSS was 2 [inter quartile range (IQR), 1–4], 30% (*n* = 95) were smokers upon study admission and 19% (*n* = 58) had a history of hypercholesterolemia. 31% (*n* = 97) had received a school education for more than 10 years. 18% (*n* = 59) had ABI ≤ 0.9. Of those, 59% (*n* = 35) had mildly low ABI (0.75 – 0.9) and 41% (*n* = 24) had moderately to severely low ABI (< 0.75). Table 1 gives a detailed overview of patient baseline characteristics.

**Table 1:** Demographic baseline characteristics according to ankle-brachial index.

	**Total**	**Normal ABI**	**Low ABI**
		**(>0.9)**	**(≤ 0.9)**
All patients, *n* (%)	325 (100)	266 (81.8)	59 (18.2)
Female sex, *n* (%)	123 (37.9)	96 (36.1)	27 (45.8)
Male sex, *n* (%)	202 (62.1)	170 (63.9)	32 (54.2)
Age in years, mean (SD)	66.1 (13.1)	65.5 (13.6)	69.0 (10.1)
Education > 10 years, *n* (%)	97 (29.8)	87 (32.7)	10 (16.9)
**Blood pressure, mm Hg, mean (SD)**
Systolic	139.9 (21.7)	139.5 (21.1)	141.8 (24.4)
Diastolic	77.5 (14.0)	78.0 (14.0)	75.1 (13.8)
History of hypertension, *n* (%)	198 (60.9)	155 (58.3)	43 (72.9)
BMI kg/m^2^ mean/median (SD)	27.5 (4.9)	27.4 (4.8)	27.7 (5.1)
< 18.5 kg/m^2^, *n* (%)	1 (0.3)	0 (0.0)	1 (1.7)
≥18.5 < 25 kg/m^2^, *n* (%)	115 (xx)	96 (35.7)	19 (31.0)
≥25– < 30 *n*, kg/m^2^, *n* (%)	129 (xx)	107 (40.3)	22 (37.9)
≥30 kg/m^2^, *n* (%)	80 (24.9)	63 (24.0)	17 (29.3)
**Cholesterol, mg/dL, mean (SD)**
Total	196.8 (47.6)	194.9 (46.3)	205.6 (52.6)
LDL	122.9 (41.5)	121.4 (39.9)	129.8 (47.9)
HDL	50.4 (15.9)	50.7 (16.1)	48.9 (14.7)
Triglycerides, mg/dL, mean (SD)	136.2 (75.2)	133.9 (72.5)	147.4 (86.9)
Blood glucose level, mean (SD)	123.2 (44.8)	124.2 (47.4)	117.0 (24.8)
Regular alcohol consumption, *n* (%)	105 (32.3)	84 (31.6)	21 (35.5)
Current smokers, *n* (%)	95 (29.2)	70 (26.3)	25 (42.3)
History of peripheral arterial disease, *n* (%)	18 (5.5)	6 (2.3)	12 (20.3)
History of diabetes mellitus, *n* (%)	65 (20.0)	53 (19.9)	12 (20.3)
History of coronary heart disease, *n* (%)	47 (14.5)	40 (15.0)	7 (11.9)
History of atrial fibrillation, *n* (%)	51 (15.7)	38 (14.3)	13 (22.0)
NIHSS at admission, median (IQR)	2 (1–4)	2 (1–4)	2 (1–4)
0–4, *n* (%)	254 (78.2)	205 (77.1)	49 (83.1)
5–16, *n* (%)	71 (21.9)	61 (22.9)	10 (17.0)
**TOAST**, ***n*** **(%)**
Large-artery atherosclerosis, *n* (%)	97 (29.9)	74 (27.8)	23 (39.0)
Cardioembolic stroke, *n* (%)	56 (17.2)	45 (16.9)	11 (18.6)
Small vessel occlusion, *n* (%)	49 (15.1)	38 (14.3)	11 (18.6)
Stroke of other determined cause, *n* (%)	11 (3.4)	9 (3.4)	2 (3.4)
Stroke of undetermined cause, *n* (%)	112 (34.5)	100 (37.6)	12 (20.3)
**Cognitive function at baseline, mean (SD)**
MMSE	27.3 (3.2)	27.5 (3.1)	26.2 (3.7)
MoCA	24.4 (4.0)	24.6 (3.9)	23.4 (4.4)
mRS at baseline, median (IQR)	2 (1–3)	2 (1–2)	2 (1–3)

SD, standard deviation; BMI, Body Mass Index; LDL, low density lipoprotein; HDL, high density protein; IQR, inter quartile range between the 25th and the 75th percentile; NIHSS, National Institutes of Health Stroke Scale; TOAST, stroke etiology according to Trial or Org 10172 in Acute Stroke Treatment; MMSE; Mini Mental State Examination (scale 0–30; cut-off score for cognitive impairment ≤ 26); MoCA; Montreal Cognitive Assessment (scale 0-30; cut-off score for cognitive impairment ≤ 25); mRS, modified Rankin Scale.

### Cognitive function at baseline

Patients with low ABI had a higher risk of baseline cognitive impairment than patients with normal ABI (MoCA adjusted RR in Model 3: 1.98; 95%-CI: 1.24 to 3.16). The risk for cognitive impairment was most pronounced in patients with moderately to severely low ABI (< 0.75) (adjusted RR for Model 3 for MoCA: 2.60; 95%-CI: 1.46 to 4.64). However, it must be noted that the MoCA was only assessed in 75% (*n* = 243) of the patients with ABI measurement. Estimates for cognitive function at baseline assessed with MoCA and MMSE are shown in the Supplementary Table 1.

### Cognitive function over time

**Figure 1:**
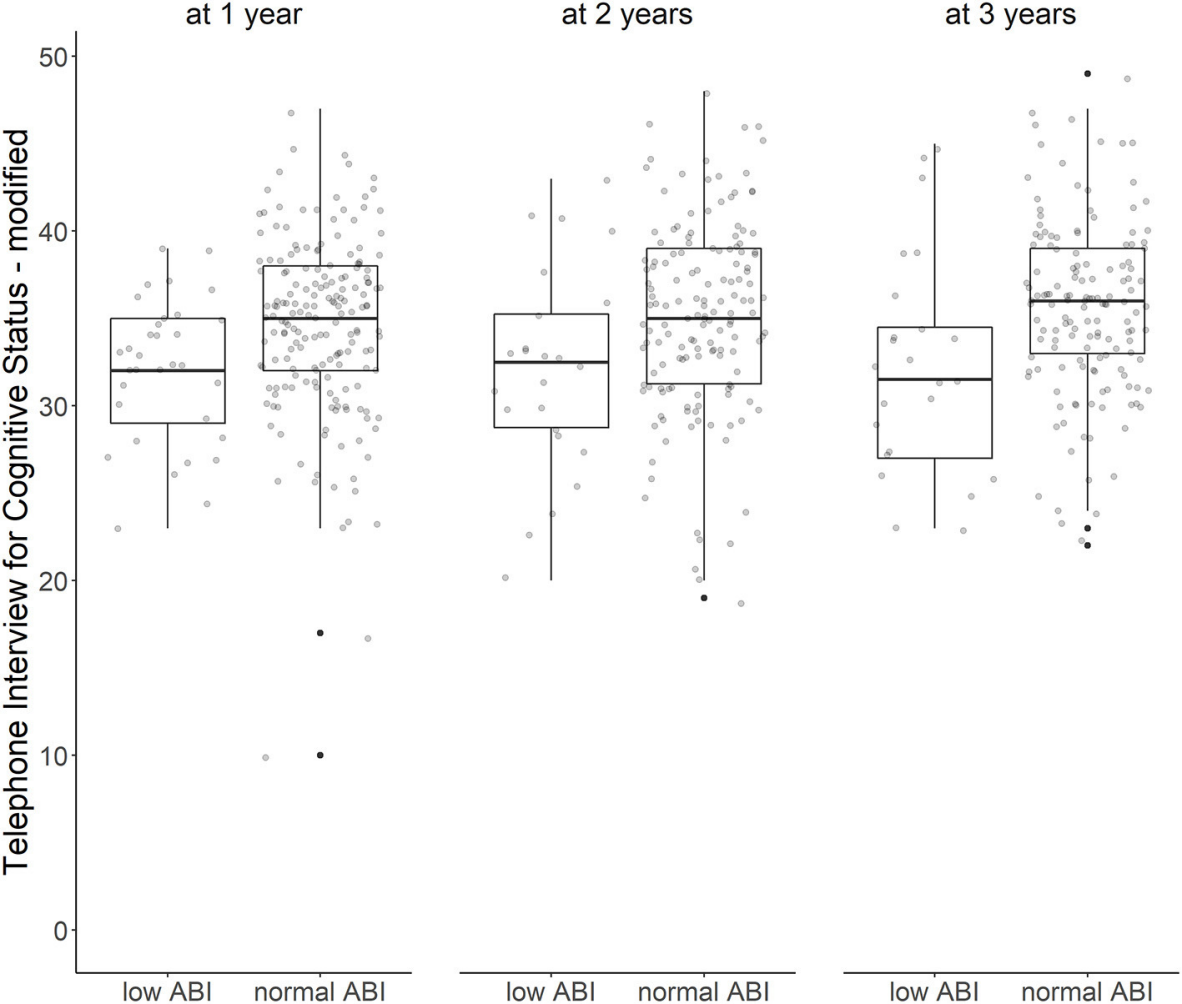
ABI and cognitive function over time. Cognitive function after first ischemic stroke with low vs. normal ankle-brachial index at 1, 2, and 3 years after stroke (Tukey's boxplots with jitter/scatterplot).

Over all follow-up time-points, patients with low ABI had lower TICS-m scores than patients with normal ABI (adjusted ß for Model 3: −1.96; 95%-CI: −3.55 to −0.37). Cognitive function was most impaired in patients with moderately to severely low ABI (adjusted ß for Model 3: −2.26; 95%-CI: −5.24 to 0.72). Cognitive function over time is illustrated in Figure 1. Cognitive function did not decline over time (adjusted ß for Model 3: 0.29; 95%-CI: −0.06 to 0.64). Results of crude and adjusted linear mixed models are shown in Table 2. Results of a sensitivity analysis stratifying low ABI can be found in Supplementary Table 2. Sensitivity analyses excluding patients who experienced a recurrent stroke or died during the three-year follow up (*n* = 44) yielded similar results (adjusted ß for Model 3: −1.90; 95%-CI: −3.57 to −0.24), as did using multiple imputation by chained events (MICE) (adjusted ß for Model 3: −1.28; 95%-CI: −2.55 to −0.01). These results are depicted in Supplementary Tables 3, 4.

**Table 2:** Association of normal (>0.9) vs. low (≤ 0.9) ABI with cognitive function based on TICS-m over a 3-year follow-up.

			Crude^*^	**Model 1^†^**	**Model 2^‡^**	**Model 3^§^**
			**ß (95%-CI)**	**ß (95%-CI)**	**ß (95%-CI)**	**ß(95%-CI)**
Model without interaction	Group effect	Change in TICS-m for normal (ABI 1.3–>0.9) (reference)	0 (ref.)	0 (ref.)	0 (ref.)	0 (ref.)
		Change in TICS-m for low ABI (≤ 0.9) vs. normal ABI (1.3–>0.9)	−2.65 (−4.28 to −1.01)	−1.83 (−3.39 to −0.26)	−1.72 (−3.32 to −0.11)	−1.96 (−3.55 to −0.37)
	Time effect	Change in TICS-m during follow-up per year	0.26 (−0.07 to 0.59)	0.29 (−0.04 to 0.62)	0.30 (−0.05 to 0.65)	0.29 (−0.06 to 0.64)

ß, effect size (represents the change in TICS-m score for each year that a patient is in follow-up after stroke); CI, confidence interval; TICS-m, telephone interview of cognitive status-modified (scale 0–50); ref., reference category.

* Crude analysis.

† Model 1, adjusted for age, sex and education.

‡ Model 2, adjusted for age, sex, education and cardiovascular risk factors (history of hypercholesterolemia, BMI, DM, smoking, history of coronary heart disease, history of atrial fibrillation).

§ Model 3, adjusted for age, sex, education, cardiovascular risk factors and stroke severity (NIHSS score).

## Discussion

The main finding of this study is that low ABI (≤ 0.9), measured after an index stroke, is associated with reduced cognitive function, both at baseline and over a 3-year follow-up compared to normal ABI. This effect is independent of age, sex, educational level and several cardiovascular risk factors and most pronounced when comparing participants with moderately to severely low ABI (< 0.75) to those with normal ABI.

Our findings expand on recent observations that low ABI is linked to cognitive impairment immediately post-stroke by demonstrating detrimental effects on cognitive function also in the long-term (5). Our results support atherosclerosis as an underlying pathomechanism for cognitive disfunction and as a contributing factor in the development of dementia (e.g., Alzheimer's disease) (14). These findings expand the use of ABI as a sensitive screening mechanism, controlling for the progression of atherosclerotic blood vessel calcification, to a biomarker for cognitive dysfunction after stroke. Research has shown that statin use helps reduce the risk of post-stroke cognitive impairment, underlining the impact of atherosclerosis as a modifiable risk factor on unfavorable stroke outcomes (15). While low ABI as a surrogate marker can be improved through protective interventions, further research is needed to examine whether modifying ABI also modifies the risk for future cognitive impairment (16).

### Strengths and limitations

Strengths of the study include its prospective design with a homogenous cohort, long follow-up period of 3 years with annual screenings and the assessment of cognitive function both at baseline and during follow-up. However, several limitations need to be addressed. First, we only included patients with mild to moderate ischemic stroke. This impedes the generalizability of our results to severe stroke patients and patients with other than ischemic stroke subtypes. However, patients who suffered severe stroke events are more likely to have low ABI at baseline and suffer from more severe cognitive impairment over time (7, 17). Secondly, predominantly male patients (62.3%) and a relatively high proportion of “strokes of undetermined cause” (35%) were included into the study. This should be taken into account when interpreting the data. Furthermore, we only assessed ABI at baseline, not during annual follow-ups. Further studies are needed to examine whether ABI is a modifiable risk factor for cognitive impairment. Thirdly, while our results showed that cognitive function was reduced both at baseline and over time in participants with low vs. normal ABI, cognitive performance did not significantly decline over time in either group. This may indicate that post-stroke cognition over time is predicted by baseline cognitive function following stroke occurrence (18). Then again, participants had only suffered mild-to-moderate ischemic strokes and may thus have remained mostly cognitively unimpaired. Moreover, the serial application of the TICS-m might have led to practice effects, masking cognitive decline (19). In addition, the cognitive tests we administered assessed global cognition, so no conclusions can be drawn as to whether the observed associations are more prominent in certain cognitive domains.

## Conclusions

In conclusion, our 3-year prospective cohort study including mild-to-moderate first-ever ischemic stroke patients showed that low ABI at stroke occurrence was associated with reduced cognitive function both at baseline and over a 3-year follow-up period. The associations were strongest in participants with moderately and severely low ABI.

## Data availability statement

The raw data supporting the conclusions of this article will be made available by the authors, without undue reservation.

## Ethics statement

The studies involving human participants were reviewed and approved by Ethics Committee Charité. The patients/participants provided their written informed consent to participate in this study.

## Author contributions

MS, BS, and TL: study conception and design. MS, PS, LB, SH, and TL: data collection. MS, SP, BS, PS, and TL: analysis and interpretation of results. MS, PS, LB, SH, SP, PH, ME, and TL: draft manuscript preparation. All authors reviewed the results and approved the final version of the manuscript.

## Funding

The PROSCIS-B study received funding from the Federal Ministry of Education and Research *via* the Grant Center for Stroke Research Berlin (01 EO 0801) up until May 2018.

## Conflict of interest

PH received research grants from the German Ministry of Research and Education, German Research Foundation, European Union, Charité, Berlin Chamber of Physicians, German Parkinson Society, University Hospital Würzburg, Robert-Koch-Institute, German Heart Foundation, Federal Joint Committee (G-BA) within the Innovationfond, Charité–Universitätsmedizin Berlin (within MonDAFIS; supported by an unrestricted research grant to the Charité from Bayer), University Göttingen (within FIND-AF-randomized; supported by an unrestricted research grant to the University Göttingen from Boehringer-Ingelheim), and University Hospital Heidelberg (within RASUNOA-prime; supported by an unrestricted research grant to the University Hospital Heidelberg from Bayer, BMS, Boehringer-Ingelheim, Daiichi Sankyo), outside submitted work. ME received grant support from Bayer, the German Research Foundation (DFG), the German Federal Ministry of Education and Research (BMBF), the German Center for Neurodegenerative Diseases (DZNE), the German Centre for Cardiovascular Research (DZHK), the European Union, Corona Foundation, and Fondation Leducq; fees paid to the Charité from Bayer, Boehringer Ingelheim, Bristol-Myers Squibb, Pfizer, Daiichi Sankyo, Amgen, GlaxoSmithKline, Sanofi, Covidien, Novartis, all outside the submitted work. The remaining authors declare that the research was conducted in the absence of any commercial or financial relationships that could be construed as a potential conflict of interest.

## Publisher's note

All claims expressed in this article are solely those of the authors and do not necessarily represent those of their affiliated organizations, or those of the publisher, the editors and the reviewers. Any product that may be evaluated in this article, or claim that may be made by its manufacturer, is not guaranteed or endorsed by the publisher.
