# Supplementary figures and images for: Low ankle-brachial index and cognitive function after stroke—the PROSpective with Incident Stroke Berlin (PROSCIS-B)
Source: Front Neurol. 2022 Sep 28;13:963262. doi: 10.3389/fneur.2022.963262 (PMC9554657; doi:10.3389/fneur.2022.963262)

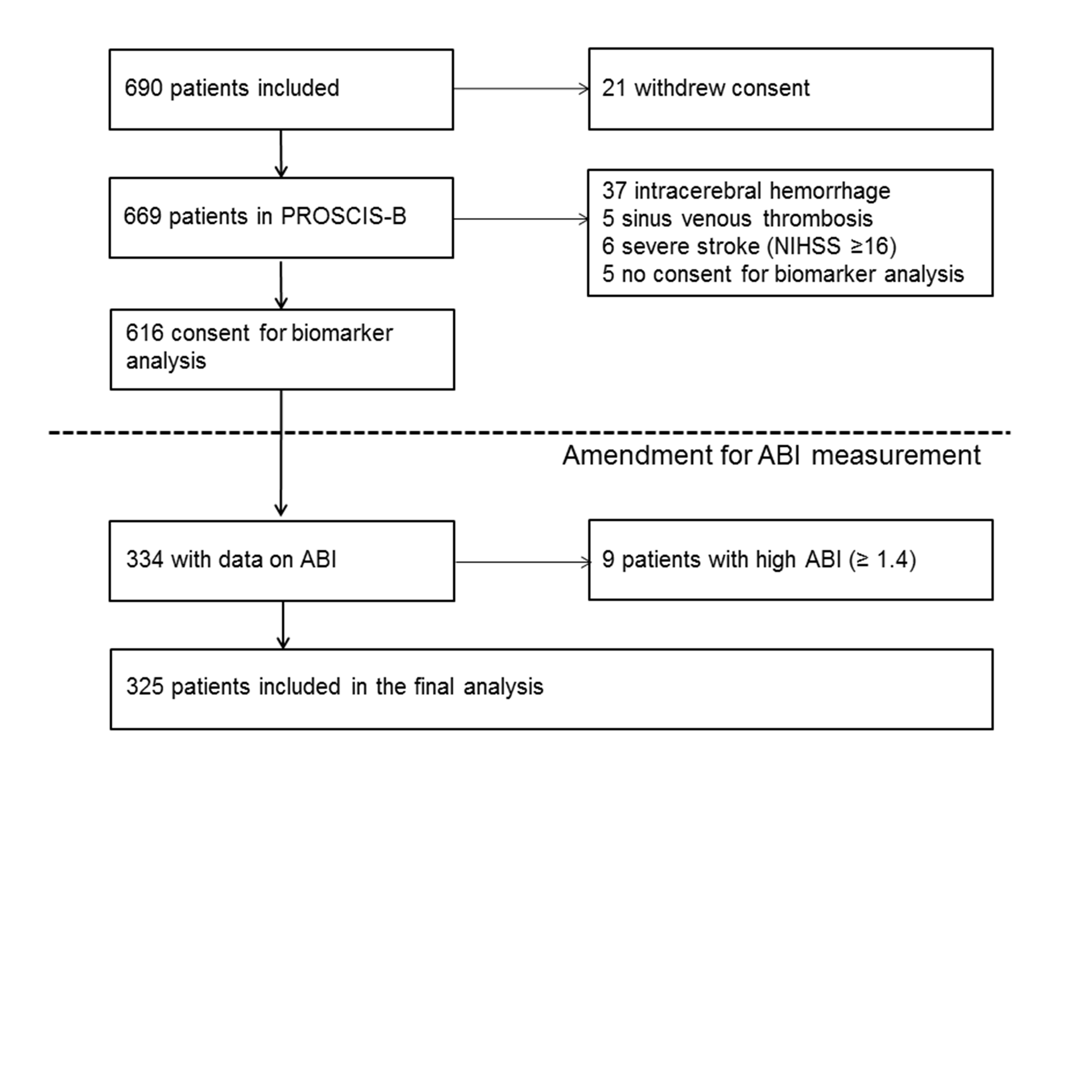

Supplement: Supplementary file 1 [file Image_1.tif]
