# Supplementary material for: Low ankle-brachial index and cognitive function after stroke—the PROSpective with Incident Stroke Berlin (PROSCIS-B)
Source: Front Neurol. 2022 Sep 28;13:963262. doi: 10.3389/fneur.2022.963262 (PMC9554657; doi:10.3389/fneur.2022.963262)
Supplement: Supplementary file 2 [file Data_Sheet_1.docx]

**SUPPLEMENTAL MATERIAL**

**Low ankle-brachial index and cognitive function after stroke – the PROspective Cohort with Incident Stroke Berlin**

Maria R.V. Stillfried et al.

**Methods I**

We conducted different sensitivity analyses. We excluded patients who had suffered a recurrent stroke or died during follow-up and recalculated the association between low versus normal ABI and cognitive function over time using the LMM to explore whether our effect estimates would remain similar. To address missing observations in our cognitive outcome measure TICS-m, we imputed missing values using multiple imputations by chained equations (MICE). We considered the following baseline variables to be predictive of the TICS-m score: age, sex, years of education, current smoking, regular alcohol consumption, history of hypertension, diabetes, hypercholesterolemia, coronary heart disease, atrial fibrillation and peripheral arterial disease, stroke type and severity according to NIHSS score and TOAST criteria, and baseline MMSE, MoCA and mRS score at baseline. We used predictive mean matching to impute continuous variables (TICS-m), logistic regression to impute dichotomous covariables and ordinal logistic regressions to impute ordinal covariables. We did not impute observations that were missing due to death. After generating ten complete datasets using MICE, we ran LMM over these 10 complete datasets and pooled resulting effect sizes according to Rubin’s rule.(1)

**References**

1. Sterne JAC, White IR, Carlin JB, Spratt M, Royston P, Kenward MG, et al. Multiple imputation for missing data in epidemiological and clinical research: Potential and pitfalls. BMJ. 2009;339(7713):157–60.

**Figure legends**

Supplemental Figure 1:

Short title: Flow-chart of patient inclusion.
Detailed legend: Flow-chart of patient inclusion. PROSCIS-B: PROSpective Cohort with Incident Stroke Berlin

**Tables:**

**Supplemental Table 1**: Association of normal vs. low ABI with cognitive impairment (MMSE ≤ 26 and MoCA ≤ 25) at baseline calculated with a GLM.

|  |  | **Crude*** | | **Model 1**^†^ | | **Model 2**^‡^ | | **Model 3**^§^ | |
| --- | --- | --- | --- | --- | --- | --- | --- | --- | --- |
|  | ABI | RR | 95% CI | RR | 95 % CI | RR | 95% CI | RR | 95% CI |
| MMSE | Normal ABI (1.4 – 0.9) | 1 | (ref.) | 1 | (ref.) | 1 | (ref.) | 1 | (ref.) |
|  | Low ABI (≤ 0.9) | 1.67 | 1.17 to 2.40 | 1.42 | 0.98 to 2.08 | 1.37 | 0.92 to 2.04 | 1.49 | 0.98 to 2.24 |
|  | *Mildly low (> 0.75 – 0.9)* | 1.55 | 0.99 to 2.45 | 1.32 | 0.80 to 2.19 | 1.29 | 0.79 to 2.09 | 1.40 | 0.85 to 2.29 |
|  | *Moderately & severely low (≤ 0.75)* | 1.86 | 1.16 to 2.98 | 1.56 | 0.97 to 2.50 | 1.50 | 0.87 to 2.60 | 1.64 | 0.95 to 2.84 |
| MoCA^\|\|^ | Normal ABI (1.4 – 0.9) | 1 | (ref.) | 1 | (ref.) | 1 | (ref.) | 1 | (ref.) |
|  | Low ABI (≤ 0.9) | 1.97 | 1.32 to 2.94 | 1.80 | 1.20 to 2.69 | 1.73 | 1.12 to 2.67 | 1.98 | 1.24 to 3.16 |
|  | *Mildly low (>0.75 – 0.9)* | 1.71 | 1.01 to 2.90 | 1.52 | 0.85 to 2.70 | 1.43 | 0.84 to 2.46 | 1.65 | 0.93 to 2.94 |
|  | *Moderately & severely low (≤ 0.75)* | 2.36 | 1.46 to 3.82 | 2.14 | 1.35 to 3.38 | 2.32 | 1.30 to 4.16 | 2.60 | 1.46 to 4.64 |

ABI, ankle-brachial index; GLM, generalized linear Model; RR, relative risk; CI, confidence interval; MMSE, Mini Mental State Examination (scale 0-30; cut-off score for cognitive impairment ≤ 26); ref., reference category; MoCA; Montreal Cognitive Assessment (scale 0-30; cut-off score for cognitive impairment ≤ 25). *Crude analysis. ^†^ Model 1, adjusted for age, sex and education. ^‡^ Model 2, adjusted for age, sex, education and cardiovascular risk factors (history of hypercholesterolemia, BMI, DM, smoking, history of coronary heart disease, history of atrial fibrillation). ^§^ Model 3, adjusted for age, sex, education, cardiovascular risk factors and stroke severity (NIHSS score).

^||^ MoCA measured in 75% (n = 243) of patients with ABI assessment.

**Table 2.** Association of stratified subgroup ABI with cognitive function based on TICS-m over a three-year follow-up.

|  |  | | **Crude*** | **Model 1**^†^ | **Model 2**^‡^ | **Model 3**^§^ |
| --- | --- | --- | --- | --- | --- | --- |
|  |  |  | ß (95%-CI) | ß (95%-CI) | ß (95%-CI) | ß (95%-CI) |
| *Model without interaction* | *Group effect* | *Change in TICS-m for normal (ABI 1.3 – >0.9)*  *(reference)* | 0 (ref.) | 0 (ref.) | 0 (ref.) | 0 (ref.) |
|  |  | *Change in TICS-m for mild low ABI (0.75 –* ≤ *0.9)* | -2.36 (-4.26 to -0.45) | -1.65 (-3.47 to 0.17) | -1.68 (-3.49 to 0.13) | -1.89 (-3.68 to -0.10) |
|  |  | *Change in TICS-m for moderately & severely low ABI (<0.75)* | -3.33 (-6.13 to -0.52) | -2.23 (-4.88 to 0.42) | -1.95 (-4.96 to 1.06) | -2.26 (-5.24 to 0.72) |
|  | *Time effect* | *Change in TICS-m during follow-up per year* | 0.26 (-0.07 to 0.59) | 0.29 (-0.04 to 0.62) | 0.30 (-0.05 to 0.65) | 0.30 (-0.05 to 0.65) |

ß, effect size (represents the change in TICS-m score for each year that a patient is in follow-up after stroke); CI, confidence interval; TICS-m, telephone interview of cognitive status-modified (scale: 0-50); ABI, ankle-brachial index. *Crude analysis. ^†^ Model 1, adjusted for age, sex and education. ^‡^ Model 2, adjusted for age, sex, education and cardiovascular risk factors (history of hypercholesterolemia, BMI, DM, smoking, history of coronary heart disease, history of atrial fibrillation). ^§^ Model 3, adjusted for age, sex, education, cardiovascular risk factors and stroke severity (NIHSS score).

**Table 3.** Association of ABI with cognitive function based on TICS-m adjusted for patients with recurrent stroke or death during the three-year follow-up.

|  |  | | | **Crude*** | **Model 1**^†^ | **Model 2**^‡^ | **Model 3**^§^ |
| --- | --- | --- | --- | --- | --- | --- | --- |
|  |  |  |  | ß (95%-CI) | ß (95%-CI) | ß (95%-CI) | ß (95%-CI) |
| *Model without interaction* | | *Group effect* | *Change in TICS-m for normal (ABI 1.3 – >0.9)*  *(reference)* | 0 (ref.) | 0 (ref.) | 0 (ref.) | 0 (ref.) |
|  | |  | *Change in TICS-m for low ABI (*≤ *0.9) vs. normal ABI (1.3 – >0.9)* | -2.36  (-4.08 to -0.63) | -1.66  (-3.30 to -0.14) | -1.58  (-3.25 to - 0.08) | -1.90  (-3.57 to -0.24) |
|  |  | *Time effect* | *Change in TICS-m during follow-up per year* | 0.28  (-0.05 to 0.61) | 0.33  (-0.01 to 0.66) | 0.34  (0.00 to 0.69) | 0.33  (-0.01 to 0.69) |

Beta, effect size (represents the change in TICS-m score for each year that a patient is in follow-up after stroke); CI, confidence interval; TICS-m, telephone interview of cognitive status-modified (scale: 0-50); ABI, ankle-brachial index. *Crude analysis. ^†^ Model 1, adjusted for age, sex and education. ^‡^ Model 2, adjusted for age, sex, education and cardiovascular risk factors (history of hypercholesterolemia, BMI, DM, smoking, history of coronary heart disease, history of atrial fibrillation). ^§^ Model 3, adjusted for age, sex, education, cardiovascular risk factors and stroke severity (NIHSS score).

**Table 4.** Association of ABI with cognitive function based on TICS-m over a three-year follow-up using multiple imputation by chained events (MICE).

|  |  | | | **Crude*** | **Model 1**^†^ | **Model 2**^‡^ | **Model 3**^§^ |
| --- | --- | --- | --- | --- | --- | --- | --- |
|  |  |  |  | Beta (95%-CI) | Beta (95%-CI) | Beta (95%-CI) | Beta (95%-CI) |
| *Model without interaction* | | *Group effect* | *Change in TICS-m for normal (ABI 1.3 – >0.9)*  *(reference)* | 0 (ref.) | 0 (ref.) | 0 (ref.) | 0 (ref.) |
|  | |  | *Change in TICS-m for low ABI (*≤ *0.9) vs. normal ABI (1.3 – >0.9)* | -1.94  (-3.32 to -0.56) | -1.29  (-2.57 to -0.01) | -1.08  (-2.36 to 0.21) | -1.28  (-2.55 to -0.01) |
|  |  | *Time effect* | *Change in TICS-m during follow-up per year* | 0.33  (-0.14 to 0.79) | 0.33  (-0.14 to 0.80) | 0.31  (-0.18 to 0.80) | 0.30  (-0.18 to 0.79) |

ß, effect size (represents the change in TICS-m score for each year that a patient is in follow-up after stroke); CI, confidence interval; TICS-m, telephone interview of cognitive status-modified (scale: 0-50); ABI, ankle-brachial index. *Crude analysis. ^†^ Model 1, adjusted for age, sex and education. ^‡^ Model 2, adjusted for age, sex, education and cardiovascular risk factors (history of hypercholesterolemia, BMI, DM, smoking, history of coronary heart disease, history of atrial fibrillation). ^§^ Model 3, adjusted for age, sex, education, cardiovascular risk factors and stroke severity (NIHSS score).
